# Trachoma or Granular Conjunctivitis

**Published:** 1907-03-16

**Authors:** Harry G. Critchley

**Affiliations:** Oculist to the London County Council Education Department


### Hospital Clinics.

### TRACHOMA OR GRANULAR CONJUNCTIVITIS.

By HARRY G. CRITCHLEY, M.A., M.D., Oculist to the London County Council Education Department.

This disease is, in this country, of comparatively rare occurrence, and it is the infrequency of its occurrence which suggests the idea of writing a short description of it, because it is so often unsuspected and overlooked; another point which suggests the idea to me is a statement, which was made three years ago, to the effect that trachoma is so common in the London schools as to interfere seriously with the administration of the Education Act. It is admitted that this is so in the New York schools, but in the London schools it is not so common as hip disease or angular curvature of the spine, and nearly all the cases are found in one area of Stepney. Searching for trachoma in consequence of the statement above referred to, we have found only 11 cases among 120,000 children, exclusive of the neighbourhood of Whitechapel and Stepney, but an inquiry directed specially to schools in these districts has revealed 14 cases among 5,000 children, all aliens, and for the most part Jews from Southern Russia and of Polish nationality.

The disease is associated with low-lying marshy countries, and with unhygienic conditions such as overcrowding, squalor, and underfeeding, and in Great Britain it occurs for the most part in the poorer Irish and Jewish communities, but it is possible that the disease may spread to districts hitherto unaffected, by reason of the alien immigrants spreading further afield than the neighbourhoods to which they have in the past so jealously limited their patronage.

### Trachoma is sometimes termed Egyptian or Military Ophthalmia, on account of it having become so disastrously epidemic in the French army in Egypt in 1798, and on the return of the armies to Europe the infection spread rapidly among the soldiers in the insanitary continental barracks.

Trachoma or granular conjunctivitis, in appearance, closely resembles follicular conjunctivitis, but otherwise there is a marked difference between them, and it is to this difference that diagnosis must be directed.

The disease is characterised by inflammation of the conjunctivæ of the lids, and the occurrence of greyish granulations of the size of a pin-head; the granulations occur principally on the upper lid and more profusely in the cul de sac where the conjunctiva is reflected from the lid on to the eyeball; while being abundant in the upper lids, they may be quite absent from the lower ones; this point is of marked importance in the matter of diagnosis, as the false granulations of follicular conjunctivitis occur more frequently and profusely on the lower lids.

The disease may be either acute or chronic; the latter may result from the former, but more commonly occurs as the primary condition, coming on insidiously and without any acute symptoms or signs, and for this reason the actual condition may miss the careful attention which it requires. An acute attack commences with swelling of the upper lid, accompanied by general conjunctivitis and marked flow of tears, inability to endure light, and much frontal pain. On everting the upper lid the granules, in mild cases, impart to the mucous membrane an appearance somewhat resembling the roughness of coarse sand-paper, but in severe cases the granules may be completely masked and the membrane be thick, red, and velvety. Sooner or later a muco-purulent or purulent secretion appears, which in favourable cases subsides, having brought about absorption of the granules in 30 or 40 days, leaving the mucous membrane smooth. More frequently the disease passes into the sub-acute or chronic form, and as the chronic form is more frequently the primary condition, it follows that it is by far the commonest form; in fact, an American observer of extensive experience goes so far as to say that acute trachoma is simply trachoma, to which an acute conjunctivitis has been added. In the chronic form there may be no more distressing symptoms than a little burning sensation and an undue feeling of weariness after application to near work, with an equally undue susceptibility to the discomfort caused by winds and dust; but the complications and sequelæ of the chronic type may be serious to a disastrous degree: the former consist of pannus and ulceration of the cornea, while the latter are distortion of the lids and altered position of the lashes.

The chronic granulations consist more or less of connective tissue which produces a cicatricial degeneration and contraction of the mucous membrane, causing inward distortion of the lids (*entropion*) and of the eyelashes (*trichiasis*) which, rubbing on the cornea, cause constant irritation, followed by inflammation and permanent opacity of the corneal conjunctiva; it also imparts to the whole lid a heavy and drooping appearance; this refers to the upper lid; while in the lower, if the granulations are plentiful, the thickened conjunctiva pushes away the lid from the globe, and this condition, assisted by the contraction of the mucous membrane, everts the lid, giving rise to *ectropion*.

### Pannus consists essentially of a new tissue growth situated underneath the epithelial layer; it is vascular, and with an abundance of cells, giving rise in the course of time to the formation of connective tissue. It commences in the upper segment of the cornea at the periphery, gradually extends towards the pupil, and is generally, though not always, confined to the upper area, thus corresponding with the most common seat of trachoma. It is stated by some to be due to the irritation and friction of the granulations on the lid, but this does not appear to hold good, because in many cases where the granulations are large and extensive, pannus may be absent; but in other cases a comparatively mild trachoma may be associated with a marked degree of pannus. If the pannus contains but little connective tissue, it may undergo a cure, the vessels becoming attenuated and then disappearing, and the opacity left behind, not being serious as its greatest density is towards the periphery; but I have never seen a pannus disappear so as to leave no opacity, as some writers state may be the case.

The disease is contagious, and is spread by the secretion; this may be conveyed by towels, sponges, handkerchiefs, hands, etc., and in hot weather by flies, and is especially prone to become epidemic in unhygienic workhouses and other institutions.

Treatment.

Frequently this is spontaneous, brought about by the incidence of an acute attack of purulent conjunctivitis, which may cause absorption of the granules, but it cannot undo any cicatrisation which may have come about.

In the acute condition, the treatment should err distinctly on the side of mildness: the employment of powerful escharotics is to be deprecated.

Firstly, the sufferer must be placed under hygienic conditions, and the conjunctivæ thoroughly cleansed half a dozen times in the 24 hours with an antiseptic lotion, such as boracic acid, 10 grains to the ounce, and once daily, the lids having been fully everted and exposed, to be painted with a solution of nitrate of silver, three grains to the ounce, together with the instillation of atropine, two grains to the ounce; under this treatment the secretion and pain should rapidly decline, but the risk of permanent staining of the conjunctiva, *argyrosis*, by the continued application of nitrate of silver, should be borne in mind, and for this reason a 10 per cent. dilution of argyrol may be preferred to the nitrate of silver.

For chronic cases the infusion of jequirity used to be recommended in the text-books. I have no experience of it beyond what I have seen in the eye clinics of the Vienna Hospital, and that has not induced me to try it. I prefer the application of sulphate of copper, a well-selected piece with a smooth surface, rubbed well into the conjunctivæ, with special attention directed to the fornices. After a time this treatment may be varied by the use of a 10 per cent. solution of silver nitrate, neutralised by the after application of a solution of salt. After the use of either of these remedies the secretion and the pain will be temporarily increased; the application should be made only on alternate days, and for the benefit of the cornea atropine should be used as in the acute condition. Before treatment cocaine should be applied.

In cases where the granulations are large and not numerous, they may be squeezed out between the thumb nails like "blackheads" in the skin. It has even been proposed, and indeed practised, to excise the fornix with its granulations. I have no experience of this form of treatment, but I am aware of the probability of a resulting scar; still, if the majority of the granules are in the fornix, the cicatrix caused by the excision should not cause so much contraction as that of the natural cicatrisation of the granulations themselves, as the scar would at any rate be linear.

The application of $x$-rays has been tried in a considerable number of cases, and while proving successful in some, seems to have unreasonably failed in an equal number.

Follicular Conjunctivitis.

This is a very common form of conjunctival inflammation, occurring more commonly in children, and characterised by minute translucent elevated bodies, resembling follicles, and anatomically so closely allied to the granules of trachoma as to be hardly distinguishable from them; but they differ from the latter in that *they are found in greater abundance in the lower lid* and arranged in rows more or less parallel with the long axis of the lid: they remain separate, and do not become confluent: *they do not cause pannus* or give rise to cicatrisation nor to drooping of the upper lid, and the secretion, if at all contagious, is not highly so.

Nevertheless I have seen typical cases of follicular conjunctivitis pointed out as being trachoma, and it is even regarded by some as being a mitigated form of trachoma; this may go far to explain the statement that trachoma is of such serious persistence in the London schools. Possibly the term "trachomatoid" conjunctivitis might not inaptly be used to describe it.

The consideration of these two diseases seems to impress upon one the necessity for the observance of a golden rule in ophthalmic practice—namely, "Never diagnose any disease associated with inflammatory condition of the conjunctiva without thoroughly everting the eyelids."

